# The active living gender’s gap challenge: 2013–2017 Eurobarometers physical inactivity data show constant higher prevalence in women with no progress towards global reduction goals

**DOI:** 10.1186/s12889-019-8039-8

**Published:** 2019-12-12

**Authors:** X. Mayo, G. Liguori, E. Iglesias-Soler, R. J. Copeland, I. Clavel San Emeterio, A. Lowe, F. del Villar, A. Jimenez

**Affiliations:** 1 Observatory of Healthy & Active Living of Spain Active Foundation, Centre for Sport Studies, King Juan Carlos University, Madrid, Spain; 2 University of Rhode Island, Kingston, RI USA; 3 Performance and Health Group, Department of Physical Education and Sport. Faculty of Sports Sciences and Physical Education, University of A Coruna, A Coruña, Spain; 4 Advanced Wellbeing Research Centre, Faculty of Health and Wellbeing, Sheffield Hallam University, Sheffield, UK; 5 The National Centre for Sport and Exercise Medicine, Sheffield, UK; 6 Galician Sport Foundation, Santiago, Spain; 7 GO fit LAB, Ingesport, Madrid, Spain

**Keywords:** Physical inactivity, Eurobarometer, Global action plan, European Union

## Abstract

**Background:**

The World Health Organization (WHO) considers physical inactivity (PIA) as a critical noncommunicable factor for disease and mortality, affecting more women than men. In 2013, the WHO set a 10% reduction of the PIA prevalence, with the goal to be reached by 2025. Changes in the 2013–2017 period of physical inactivity prevalence in the 28 European Union (EU) countries were evaluated to track the progress in achieving WHO 2025 target.

**Methods:**

In 2013 and 2017 EU Special Eurobarometers, the physical activity levels reported by the International Physical Activity Questionnaire of 53,607 adults were analyzed. Data were considered as a whole sample and country-by-country. A χ2 test was used to analyze the physical inactivity prevalence (%) between countries, analyzing women and men together and separately. Additionally, PIA prevalence was analyzed between years (2013–2017) for the overall EU sample and within-country using a *Z*-Score for two population proportions.

**Results:**

The PIA prevalence increased between 2013 and 2017 for the overall EU sample (*p* <  0.001), and for women (*p* = 0.04) and men (*p* < 0.001) separately. Data showed a higher PIA prevalence in women versus men during both years (*p* <  0.001). When separately considering changes in PIA by gender, only Belgium’s women and Luxembourg’s men showed a reduction in PIA prevalence. Increases in PIA prevalence over time were observed in women from Austria, Croatia, Germany, Lithuania, Malta, Portugal, Romania, and Slovakia and in men from Bulgaria, Croatia, Czechia, Germany, Italy, Lithuania, Portugal, Romania, Slovakia, and Spain.

**Conclusions:**

PIA prevalence showed an overall increase across the EU and for both women and men between 2013 and 2017, with higher rates of PIA reported for women versus men during both years. PIA prevalence was reduced in only Belgium’s women and Luxembourg’s men. Our data indicate a limited gender-sensible approach while tacking PIA prevalence with no progress reaching global voluntary reductions of PIA for 2025.

## Background

Physical inactivity (PIA) is a global risk factor for disease and mortality, which is defined as individuals not meeting the weekly *Global Recommendations* on physical activity [1]. The physical activity recommendations aim to provide guidance through primary prevention on the dose-response relationship between physical activity and health benefits and address the links between frequency, duration, intensity, type and the total amount of physical activity needed for the prevention of non-communicable diseases [1]. In the World Health Organization (WHO) European region, PIA is the attributable risk factor for 12.% of the type 2 diabetes, 8% of the colon cancers, and 9.7% of all-cause mortality anually. This burden represents a lifetime disease of 2.270 disability-adjusted life-years [2]. Accordingly, conservative analyses have reported direct and indirect annual health-care costs of $11.743 and $3.829 million, respectively, for the European region [2].

The *Global Action Plan* (2013) positioned PIA as one of the critical noncommunicable diseases factors, and set for all countries a PIA reduction of 10% by 2025, relative to each country’s baseline [3]. For this mandate, member states were expected to develop national targets and indicators based on the global monitoring framework [3]. Concurrently, member states had to link this framework with a multisectoral policy represented in national plans [3]. Since PIA prevalence is consistently higher in women than in men [4–6], each country framework was intended to consider gender-based approaches to decreasing PIA, in an effort to reduce the risks of morbidity and mortality from non-communicable diseases [3]. Therefore, monitoring current levels and trends of PIA prevalence with a gender-specific approach is crucial to analyze any progress towards the goal of reduced PIA prevalence [6]. Although there is limited availability of objectively measured physical activity data (i.e., obtained by accelerometry) at present, monitoring is possible based on sex-disaggregated self-report data [7].

Regarding the European Union (EU), after considering the WHO resolutions WHA51.17 (2000) and EB109/14 (2001), systematic surveys in its member states were carried out since 2002 to monitor levels of PIA prevalence with self-report data gathered from the short form of the International Questionnaire of Physical Activity (IPAQ) [8]. During the same years, scientific papers analyzing the PIA prevalence of particular Eurobarometers were published, such as the Special Eurobarometer 183.6 (2002) [9] and the Special Eurobarometer 412 (2012) [10]. A further study observed PIA prevalence reductions for those European countries that joined the EU before 2004 comparing the Special Eurobarometer 412 and the Special Eurobarometer 246 between 2002 and 2005 [11]. Nevertheless, the picture is quite different in the 28-country EU nowadays. In this regard, a recent pooled analysis including available worldwide data through the 2013 Special Eurobarometer observed a gradual increase of PIA prevalence in Central and Eastern Europe and high-income Western countries [6].

Relevant to this matter, the publication of the *Global Action Plan* (2013) coincided with the fieldwork of the Special Eurobarometer 412, performed in late 2013. As an outcome of the *Global Action Plan*, many countries have now adopted national plans in different political domains such as sustainable environment, public health, sports promotion or active transport and in different settings such as school- or work-related activity, all aimed at reducing PIA prevalence [12]. Importantly here, most of these containing gender-specific interventions [13]. Whilst the adoption of national plans to promote physical activity is encouraging, questions do exist as to the quality and consistency of implementation [12]. With this in mind, the 2018 publication of the new Special Eurobarometer 472 regarding *Sport and Physical Activity* seems relevant to analyze possible changes in PIA prevalence in the most recent period, 2013–2017. This analysis will help determine if any changes in PIA prevalence have occurred towards the 2025 target of a 10% PIA reduction. Given the importance of gender-specific interventions to tacking PIA in women and in order to check progress and identify potential challenges, a particular focus on the women’s PIA prevalence changes is warranted [3].

This analysis aimed to track changes in PIA prevalence between 2013 and 2017 in the 28 EU countries, analyzing the respective *Sport and Physical Activity* Eurobarometer’s data. For this, we analyzed the prevalence of PIA considering the between-country differences for both years and the changes within-country between years. Our analysis explored the sample as a whole and split by gender. Our study assesses the progress in the fulfillment of the 10% reduction of PIA prevalence for 2025. It also provides comparison of the changes in PIA prevalence in individual countries and the EU as a whole against the suitability of policy action on PIA emerged from the *Physical activity strategy for the WHO European Region 2016–2025* [14] and the *Global action plans* on physical activity for the periods 2013–2020 and 2018–2030 [3, 15].

## Methods

### Data source

In the EU, public opinion surveys are conducted recurrently and simultaneously on all state members by the European Commission to inquire about physical activity and sports participation among its citizens. These surveys were conducted in 2002, 2005, 2009, 2013, and 2017 through the *Sport and Physical Activity* and *Health and Food* Special Eurobarometers.

For this analysis, data from two successive Eurobarometer surveys were obtained, December 2013 (Special Eurobarometer 412, published on March 2014, *n* = 27,919) [16] and December 2017 (Special Eurobarometer 472, published on March 2018, *n* = 28,031) [17]. The total sample (*n* = 55,950) was considered from the 28 EU member countries (Austria, Belgium, Bulgaria, Croatia, Cyprus, Czechia, Denmark, Estonia, Finland, France, Germany [combined West and East Deutschland], Greece, Hungary, Ireland, Italy, Latvia, Lithuania, Luxembourg, Malta, Netherlands, Poland, Portugal, Romania, Slovakia, Slovenia, Spain, Sweden, and United Kingdom), and Northern Ireland was not considered due to its unique characteristics. Although Eurobarometers respondents are aged 15 and over, only respondents over the age of 18 were analyzed since the physical activity recommendations are different for individuals under 18 years [1].

Eurobarometers use a multi-stage, random sampling design. For this, the number of sampling points was drawn with probability proportional to population size and population density, covering the whole territory of each country. For the sampling, a comparison between the sample and each country population was carried out. In this regard, gender, age, region, and the size of the locality were introduced in the iteration procedure. All interviews are conducted face-to-face in people’s homes and in the appropriate national language [16, 17]. Since our analysis was performed considering data from a public repository, our study was carried out addressing STROBE guidelines when possible [18].

### Measures

A modified version of the short form of the IPAQ was used to determine the prevalence of PIA [8]. The IPAQ measures the intensity, frequency, and duration of the physical activity performed in the last seven days. This information was obtained by the questions inquiring about the number of days practicing vigorous and moderate physical activity and walking activity and their respective minutes during those days. In the 2013 and 2017 Special Eurobarometers, answers were truncated to five different fixed possibilities instead of the classical open solution to indicate the minutes performed in every activity [8]. In this regard, a response of “30 minutes or less” was assumed to mean 15 min, a response of “31 to 60 minutes” was assumed to mean 45 min, a response of “61 to 90 minutes” was assumed to mean 75 min, a response of “91 to 120 minutes” was assumed to mean 105 min, and a response of “more than 120 minutes” was assumed to mean 120 min [10]. The instructions of the November 2005 version of the Guidelines for data processing and analysis of the IPAQ short form were used for analyzing the data [8]. This analysis was implemented using a modified ad hoc spreadsheet available online [19]. Only individuals with at least one valid intensity and duration of a particular intensity (i.e., both variables with a different answer than “don’t know”) were eligible for further analysis [11].

The Guidelines assume that vigorous intensity, moderate intensity, and walking represent 8.0, 4.0, and 3.3 metabolic equivalents (MET), respectively [8]. Vigorous, moderate, and walking MET-minutes/week are calculated from multiplying the selected MET values by the minutes and by the days of each intensity. Thus, the total physical activity MET-minutes/week is calculated summing up the vigorous, moderate, and walking MET-minutes/week scores.

In this regard, individuals are considered physically active when performing (a) at least 3 days of vigorous intensity activity of at least 20 min per day, (b) at least 5 days of moderate intensity activities and/or walking for at least 30 min per day, or (c) at least 5 days combining the aforementioned intensities achieving at least 600 MET-minutes/week. Individuals not reaching any of those thresholds were considered to have a “low” physical activity level, thus being classified as physically inactive.

### Statistical analysis

The PIA prevalence between countries, entire sample and separately for gender, were analyzed with a χ2 test for both 2013 and 2017. Additionally, the PIA prevalence was analyzed between both years (2013 and 2017) for the overall EU sample and within-country (Austria, Belgium, Bulgaria, Croatia, Cyprus, Czechia, Denmark, Estonia, Finland, France, Germany [combined West and East Deutschland], Greece, Hungary, Ireland, Italy, Latvia, Lithuania, Luxembourg, Malta, Netherlands, Poland, Portugal, Romania, Slovakia, Slovenia, Spain, Sweden, and United Kingdom), also as an entire sample and separetly by gender, and using a *Z*-Score for two population proportions. Data are represented as a percentage (%) with a 95% confidence interval (95% CI). A priori alpha level was set at 0.05. Statistical analyses were carried out using Microsoft Excel version 1709 (Microsoft Corporation; Redmond, Washington, United States of America).

## Results

Between the 28-country sample, significant differences in the prevalence of PIA were observed in 2013 (*n* = 26,507; χ^2^ = 1437,328; DF = 27; *p* <  0.001) and 2017 (*n* = 27,100; χ^2^ = 1643,243; DF = 27; *p* <  0.001). In the same way, significant differences between countries were also observed in the prevalence of PIA for women in 2013 (*n* = 14,503; χ^2^ = 1006,693; DF = 27; *p* <  0.001) and 2017 (*n* = 14,873; χ^2^ = 1050,121; DF = 27; *p* <  0.001) and for men in 2013 (*n* = 12,004; χ^2^ = 481,643; DF = 27; *p* <  0.001) and 2017 (*n* = 12,227; χ^2^ = 649,808; DF = 27; *p* <  0.001).

When comparing the prevalence of PIA between 2013 and 2017 (Table 1), PIA increased between years for the overall EU sample. Not all the countries experienced increases in PIA prevalence. In this sense, 17 countries maintained their PIA prevalence (Belgium, Cyprus, Denmark, Estonia, Finland, France, Greece, Hungary, Ireland, Italy, Latvia, Luxembourg, Netherlands, Poland, Slovenia, Sweden, and United Kingdom). Conversely, 11 countries reported increases in PIA prevalence between years (Austria, Bulgaria, Croatia, Czechia, Germany, Lithuania, Malta, Portugal, Romania, Slovakia, and Spain). No reductions were observed for any countries between such years.

**Table 1:** Prevalence (expressed as a percentage) of adults not complying with the World Health Organization’s aerobic physical activity recommendations in the European Union countries between 2013 and 2017

	2013		2017		*Z*-score	*p*-value
	Prevalence (%)	95% CI	Prevalence (%)	95% CI		
European Union (*n* = 53,607)	38.1%	38.4–39.5%	41.7%	41.1–42.3%	8.43	< 0.001*
Country						
Austria (*n* = 2014)	36.1%	33.1–39.0%	43.6%	40.5–46.6%	3.44	< 0.001*
Belgium (*n* = 2029)	46.4%	43.3–49.4%	44.5%	41.7–47.2%	0.86	0.39
Bulgaria (*n* = 1992)	41.1%	38.0–44.2%	49.3%	46.2–52.4%	3.68	< 0.001*
Croatia (*n* = 2007)	32.3%	29.4–35.2%	45.9%	42.9–49.0%	6.24	< 0.001*
Cyprus (*n* = 969)	64.6%	60.3–68.9%	62.1%	57.8–66.5%	0.79	0.43
Czechia (*n* = 2004)	37.3%	34.3–40.3%	42.7%	39.7–45.8%	2.48	0.01*
Denmark (*n* = 1986)	30.5%	27.6–33.4%	31.4%	28.5–34.3%	0.44	0.66
Estonia (*n* = 1967)	31.4%	28.5–34.3%	29.7%	26.8–32.6%	−0.83	0.41
Finland (*n* = 1955)	29.1%	26.2–32.0%	29.9%	27.1–32.7%	0.39	0.69
France (*n* = 1992)	42.6%	39.5–45.6%	45.0%	41.9–48.1%	1.10	0.27
Germany (*n* = 2792)	19.4%	17.2–21.6%	25.9%	23.7–28.1%	4.05	< 0.001*
Greece (*n* = 1952)	48.1%	45.0–51.2%	50.6%	47.4–53.7%	1.09	0.28
Hungary (*n* = 2026)	42.6%	39.6–45.7%	42.2%	39.2–45.2%	−0.21	0.84
Ireland (*n* = 1959)	37.9%	34.9–41.0%	42.2%	39.1–45.3%	1.95	0.051
Italy (*n* = 2011)	58.5%	55.4–61.6%	62.4%	59.4–65.4%	1.79	0.07
Latvia (*n* = 1948)	25.7%	22.9–28.4%	28.6%	25.7–31.4%	1.44	0.15
Lithuania (*n* = 1967)	33.9%	31.0–36.9%	44.5%	41.4–47.5%	4.77	< 0.001*
Luxembourg (*n* = 970)	33.9%	29.7–38.1%	29.1%	25.0–33.2%	−1.62	0.10
Malta (*n* = 988)	62.0%	57.7–66.3%	70.9%	66.9–74.9%	2.94	< 0.001*
Netherlands (*n* = 2004)	23.9%	21.2–26.5%	24.3%	21.6–26.9%	0.21	0.83
Poland (*n* = 1905)	55.3%	52.2–58.5%	53.2%	50.1–56.4%	−0.92	0.35
Portugal (*n* = 2086)	59.3%	56.2–62.3%	68.2%	65.4–71.1%	4.27	< 0.001*
Romania (*n* = 1941)	34.3%	31.3–37.3%	50.4%	47.2–53.5%	7.15	< 0.001*
Slovakia (*n* = 2038)	37.6%	34.5–40.6%	45.6%	42.6–48.6%	3.68	< 0.001*
Slovenia (*n* = 2112)	39.4%	36.5–42.3%	37.2%	34.2–40.2%	−1.04	0.30
Spain (*n* = 1989)	28.4%	25.6–31.3%	34.0%	31.0–36.9%	2.66	0.01*
Sweden (*n* = 2024)	22.0%	19.4–24.6%	23.0%	20.5–25.6%	0.56	0.57
United Kingdom (*n* = 1980)	40.6%	37.5–43.7%	37.1%	34.1–40.0%	−1.60	0.11

*CI* Confidence intervals

*Significant increases in physical inactivity prevalence (*p* ≤ 0.05)

When analyzing gender differences (Table 2), the prevalence of PIA in the overall EU sample was higher in women compared to men in both 2013 and 2017. However, rates of in-country PIA prevalence between genders varied by country for both years. For 2013 year, women had higher PIA prevalence than men in Belgium, Croatia, Cyprus, Czechia, France, Greece, Hungary, Ireland, Italy, Malta, Poland, Portugal, Slovenia, Spain, and the United Kingdom. For 2017, women had a higher prevalence of PIA for Austria, Cyprus, France, Greece, Ireland, Malta, Portugal, Spain, and United Kingdom in comparison with men.

**Table 2:** Prevalence (expressed as a percentage) of men and women not complying with the World Health Organization’s aerobic physical activity recommendations in the European Union countries between 2013 and 2017 and differences in the prevalence between both genders and for the same years

	Gender (sample)	2013				2017				2013–2017	
		Prevalence (%)	95% CI	Z-score	*p*-value	Prevalence (%)	95% CI	Z-score	*p*-value	Z-score	*p*-value
European Union	Women	42.1%	41.3–42.9%	11.75	< 0.001*	43.8%	43.0–44.6%	7.83	< 0.001*	2.88	0.04*
	Men	35.1%	34.2–35.9%			39.1%	38.2–40.0%			6.48	< 0.001*
Country-by-country											
Austria	Women (*n* = 1062)	37.1%	32.9–41.2%	0.69	0.49	46.5%	42.3–50.8%	2.01	0.04#	3.13	0.002*
	Men (*n* = 952)	34.9%	30.7–39.2%			40.3%	35.9–44.7%			1.69	0.09
Belgium	Women (*n* = 1057)	53.9%	49.7–58.1%	5.04	< 0.001#	46.8%	42.8–50.8%	1.57	0.12	**−2.30**	**0.02&**
	Men (*n* = 972)	38.3%	34.1–42.6%			41.8%	38.0–45.6%			1.11	0.27
Bulgaria	Women (*n* = 1076)	42.9%	38.7–47.1%	1.22	0.22	48.6%	44.5–52.8%	−0.47	0.64	1.89	0.06
	Men (*n* = 916)	39.0%	34.6–43.5%			50.1%	45.5–54.7%			3.37	< 0.001*
Croatia	Women (*n* = 1128)	35.5%	31.5–39.5%	2.40	0.02#	44.8%	40.7–48.9%	−0.82	0.41	3.18	0.001*
	Men (*n* = 879)	28.3%	24.1–32.5%			47.4%	42.7–52.1%			5.83	< 0.001*
Cyprus	Women (528)	70.5%	65.0–76.1%	2.93	0.003#	69.6%	64.1–75.1%	3.81	< 0.001#	−0.23	0.82
	Men (*n* = 441)	57.8%	51.3–64.2%			52.8%	46.1–59.4%			−1.06	0.29
Czechia	Women (*n* = 1157)	40.8%	36.8–44.8%	2.72	0.006#	43.5%	39.5–47.6%	0.60	0.55	0.93	0.35
	Men (*n* = 847)	32.4%	27.9–36.9%			41.6%	37.0–46.3%			2.79	0.01*
Denmark	Women (*n* = 993)	28.6%	24.7–32.5%	−1.33	0.18	29.8%	25.8–33.9%	−1.06	0.29	0.43	0.67
	Men (*n* = 993)	32.5%	28.3–36.7%			32.9%	28.9–37.0%			0.15	0.88
Estonia	Women (*n* = 1227)	33.4%	29.6–37.2%	1.64	0.10	29.8%	26.3–33.4%	0.12	0.90	−1.34	0.18
	Men (*n* = 740)	28.5%	24.0–32.9%			29.4%	24.6–34.3%			0.29	0.77
Finland	Women (*n* = 1036)	28.9%	25.0–32.8%	−0.17	0.87	29.4%	25.5–33.4%	−0.34	0.74	0.20	0.84
	Men (*n* = 919)	29.4%	25.1–33.7%			30.4%	26.3–34.5%			0.34	0.73
France	Women (*n* = 1098)	50.2%	46.0–54.4%	5.28	< 0.001#	50.9%	46.7–55.0%	4.23	< 0.001#	0.24	0.81
	Men (*n* = 894)	33.6%	29.3–37.9%			37.4%	32.9–42.0%			1.18	0.24
Germany	Women (*n* = 1396)	19.7%	16.6–22.9%	0.29	0.77	26.5%	23.4–29.6%	0.54	0.59	2.94	0.003*
	Men (*n* = 1396)	19.1%	16.0–22.1%			25.3%	22.2–28.4%			2.77	0.006*
Greece	Women (*n* = 1043)	51.6%	47.2–55.9%	2.26	0.02#	56.1%	51.9–60.3%	3.79	< 0.001#	1.45	0.15
	Men (*n* = 909)	44.3%	39.8–48.8%			43.9%	39.3–48.5%			−0.13	0.9
Hungary	Women (*n* = 1191)	47.7%	43.6–51.7%	3.85	< 0.001#	43.9%	39.9–47.8%	1.34	0.18	−1.32	0.19
	Men (*n* = 835)	35.4%	30.8–40.1%			39.7%	35.1–44.4%			1.28	0.2
Ireland	Women (*n* = 1062)	42.6%	38.5–46.8%	3.44	< 0.001#	47.6%	43.2–51.9%	3.55	< 0.001#	1.62	0.11
	Men (*n* = 897)	31.9%	27.4–36.3%			36.4%	32.0–40.7%			1.43	0.15
Italy	Women (*n* = 1065)	64.6%	60.6–68.6%	4.38	< 0.001#	65.0%	60.9–69.1%	1.72	0.09	0.13	0.9
	Men (*n* = 946)	50.8%	46.1–55.5%			59.8%	55.5–64.0%			2.27	0.01*
Latvia	Women (*n* = 1144)	28.0%	24.2–31.8%	1.83	0.07	30.2%	26.6–33.9%	1.51	0.13	0.82	0.41
	Men (*n* = 804)	22.9%	19.0–26.8%			25.7%	21.2–30.2%			0.93	0.35
Lithuania	Women (*n* = 1162)	36.0%	31.9–40.1%	1.50	0.13	45.8%	41.9–49.7%	1.15	0.25	3.40	< 0.001*
	Men (*n* = 805)	31.4%	27.1–35.8%			42.1%	37.0–47.1%			3.13	< 0.001*
Luxembourg	Women (*n* = 579)	33.2%	27.8–38.6%	−0.41	0.68	32.1%	26.7–37.5%	1.73	0.08	−0.30	0.77
	Men (*n* = 391)	35.0%	28.4–41.7%			24.7%	18.7–30.8%			**−2.22**	**0.03&**
Malta	Women (*n* = 582)	66.3%	60.9–71.7%	2.39	0.02#	76.7%	71.9–81.6%	3.37	< 0.001#	2.79	0.01*
	Men (*n* = 406)	55.6%	48.7–62.6%			62.9%	56.3–69.4%			1.49	0.14
Netherlands	Women (*n* = 1013)	23.5%	20.0–27.1%	−0.26	0.79	26.2%	22.2–30.2%	1.37	0.17	0.99	0.32
	Men (*n* = 991)	24.2%	20.3–28.2%			22.5%	19.0–26.1%			−0.64	0.52
Poland	Women (*n* = 1115)	58.1%	54.1–62.1%	2.19	0.03#	52.6%	48.5–56.7%	−0.45	0.64	−1.88	0.06
	Men (*n* = 750)	50.8%	45.7–56.0%			54.1%	49.2–59.1%			0.90	0.37
Portugal	Women (*n* = 1197)	65.5%	61.6–69.4%	4.54	< 0.001#	72.7%	69.2–76.2%	3.74	< 0.001#	2.69	0.01*
	Men (*n* = 889)	51.5%	47.0–56.1%			61.8%	57.2–66.4%			3.08	< 0.001*
Romania	Women (*n* = 992)	36.6%	32.3–40.9%	1.47	0.14	51.7%	47.3–56.0%	0.86	0.39	4.78	< 0.001*
	Men (*n* = 949)	32.1%	28.0–36.2%			48.9%	44.3–53.5%			5.28	< 0.001*
Slovakia	Women (*n* = 1161)	37.9%	33.9–41.9%	0.29	0.77	44.6%	40.6–48.5%	−0.79	0.43	2.29	0.02*
	Men (*n* = 877)	37.0%	32.4–41.7%			47.0%	42.4–51.5%			2.97	< 0.001*
Slovenia	Women (*n* = 1194)	42.6%	38.8–46.4%	2.55	0.01#	39.6%	35.5–43.7%	1.73	0.08	1.05	0.30
	Men (*n* = 918)	34.9%	30.6–39.3%			34.3%	30.0–38.7%			−0.19	0.85
Spain	Women (*n* = 1079)	32.4%	28.4–36.4%	2.92	0.003#	37.2%	33.2–41.2%	2.39	0.02#	1.66	0.10
	Men (*n* = 910)	24.0%	20.1–27.9%			30.0%	25.7–34.2%			2.04	0.04
Sweden	Women (*n* = 978)	23.4%	19.6–27.1%	1.02	0.31	22.4%	18.8–26.1%	−0.43	0.67	−0.34	0.73
	Men (*n* = 1046)	20.7%	17.1–24.2%			23.6%	20.0–27.1%			1.13	0.26
United Kingdom	Women (*n* = 1021)	44.0%	39.7–48.3%	2.28	0.02#	40.6%	36.4–44.9%	2.36	0.02#	−1.08	0.28
	Men (*n* = 959)	36.8%	32.3–41.2%			33.5%	29.3–37.6%			−1.07	0.29

*CI* Confidence intervals

*Significant increases in physical inactivity prevalence (*p* ≤ 0.05)

&Significant reductions in physical inactivity prevalence (*p* ≤ 0.05)

#Significant higher prevalence of physical inactivity in women in comparison with men (*p* ≤ 0.05)

When analyzing the subsamples of women and men separately, increases in PIA prevalence also varied by country and by year. Particularly, increases in PIA prevalence for women between 2013 and 2017 were observed for Austria, Croatia, Germany, Lithuania, Malta, Portugal, Romania, and Slovakia. Reductions in PIA prevalence for woman were only noted in Belgium. For men, increases in the PIA prevalence between 2013 and 2017 were observed for Bulgaria, Croatia, Czechia, Germany, Italy, Lithuania, Portugal, Romania, Slovakia, and Spain. Reductions in men PIA prevalence were only observed in Luxembourg.

## Discussion

The main findings of this study were: (a) the PIA prevalence increased between 2013 and 2017 for the overall EU sample and both women and men separately; (b) a higher prevalence of PIA was observed in women for both 2013 and 2017 in comparison with men; (c) reductions in PIA prevalence were only observed in Belgian women and Luxembourg men; and (d) increases in PIA prevalence were observed for women in Austria, Croatia, Germany, Lithuania, Malta, Portugal, Romania, and Slovakia, and for men in Bulgaria, Croatia, Czechia, Germany, Italy, Lithuania, Portugal, Romania, Slovakia, and Spain. In summary, reductions were rare and increases were common regarding PIA prevalence for both women and men.

To the best of our knowledge, this is the first study reporting data from the Special Eurobarometer 472, the most current dataset regarding physical activity data for EU countries (2018). Although a previous study reported data regarding the Special Eurobarometer 412 [10], there are two important differences to consider when comparing findings here with those reported previously. Firstly, Gerovasili et al. study [10] characterized the physically inactive individuals based on the total minutes performed in vigorous and moderate activity, with walking considered a moderate activity. Our study used the IPAQ Guidelines for data processing and analysis, and considered a “low” physical activity as being physically inactive, and also discerned between moderate activity and walking [8]. Gerovasili and colleagues also only analyzed physical activity among adults between 18 to 64 years old, yet our analysis consisted of adults from 18 and older (i.e., without an upper limit) since the WHO recommendations are virtually the same for aerobic physical activity regardless of upper age [1]. These two factors could account for the lower prevalence of PIA in Gerovasili et al. and should be taking into consideration when comparing the data [10].

Only one previous study compared changes between years in the prevalence of PIA using the Special Eurobarometer data between 2002 and 2005 [11]. In this study, Mayo and colleagues showed a reduction of PIA prevalence between years with dissimilar changes between countries [11]. In this regard, there may be relatively higher PIA prevalence in our study due to several reasons. Firstly, it is essential to note that in Mayo’s study only the fifteen countries that entered the EU before May 2014 were analyzed (i.e., Austria, Belgium, Denmark, Finland, France, Germany Greece, Ireland, Italy, and Luxemburg) [11], and some of these being countries with historically lower PIA prevalence [10]. Our study included all 28 EU countries, with some countries having a relatively higher prevalence of PIA, such as Cyprus or Malta [10]. Secondly, the responses in the Mayo study were the classical open solution to indicate minutes performed in every activity [11], as is indicated in the IPAQ Guidelines [8]. However, the last two Special Eurobarometers (i.e., 2013 and 2017) truncated the possible answers to five different fixed possibilities [8]. This truncation will tend to increase the replicability of the data due to a narrowed range of possibilities to answer [20, 21]. Additionally, it will reduce the minutes reported as a consequence of creating an artificial average. This false average will tend to result in higher levels of PIA in comparison with previous years [10, 11]. In this regard, a standardized instrument throughout the years to remove the limiting comparability of these survey data when using the IPAQ is needed, as was previously pointed out [6]. There have been previous attempts to standardize instruments, questions, and ways to report results in European surveys, but with a limited implementation success in the delivery [22].

As was previously explained, our data cannot be directly compared with that of previous reports, but changes observed between both reports (2002 vs. 2005 and 2013 vs. 2017) are potentially comparable since each shared the same response characteristics [11]. In this sense, there was a general reduction in the PIA prevalence in Mayo et al. for the whole sample and women and men separately [11], while in this analysis the PIA prevalence of the whole sample and for women and men increased. In particular, for the sample of every country of the fifteen analyzed in the previous report [11], none reduced the PIA prevalence in the 2013–2017 period. In this 15-country sample, four possible cases happened: Firstly, Austria, Germany, and Sweden reduced the PIA prevalence in the period 2002–2005 but increased such prevalence in the period 2013–2017. Secondly, Portugal and Spain did not change the PIA prevalence in the 2002–2005 but increased such prevalence in the 2013–2017 period. Thirdly, Belgium, France, Greece, Netherlands, and the United Kingdom did reduce the PIA prevalence in the period 2002–2005, but without showing changes in the 2013–2017 one. Lastly, Denmark, Finland, Ireland, Italy, and Luxembourg did not change the PIA prevalence in any of both periods.

Globally, our data agree with a pooled analysis of 358 population-based surveys performed through 2016, in which the PIA prevalence in Central and Eastern Europe and high-income Western countries gradually increased [6]. In this study, data were analyzed until 2013 Special Eurobarometer, including some country-specific surveys until 2016 (e.g., Germany). Since our data show increases until 2017, this suggests no progress in reducing the PIA prevalence to reach the 2025 global 10% reduction target [3]. As previously indicated by the Bangkok Declaration, our data recognize that previous effort to decrease PIA prevalence to reach such global reduction target has been insufficient [23]. This consideration points out the urgent need to strengthen policy action [23], following the objectives proposed by the new *Global Action Plan* and starting to work in a new framework with 20 policy actions within four strategic objectives [15].

When considering gender, reductions in PIA prevalence in the 2013–2017 period were only observed in the subsample of Belgium’s women and Luxembourg’s men. While to find direct causations when reducing PIA is challenging to bring in, particular interventions and actions influencing this behavior are possible to be described. For women [6], reductions of PIA prevalence in Belgium’s women were also observed in the 2002–2005 period [11]. As an example, a scientifically analyzed wide-scale intervention in Flanders (i.e., ‘10,000 Steps Flanders’) showed high levels of awareness, adoption, and implementation [24], while effectively reducing PIA prevalence with enduring effects throughout years [25]. On the other hand, physical activity-related health promotion campaigns, which are required to be freely broadcasted by law, have been performed in Wallonia region in both public and private television and radio channels [26]. This all suggests a defined policy to tackle PIA, while showing a genuine interest in tacking PIA at a policy level within those years [27]. Nevertheless, some issues in leadership and coordination at the national and sub-national level (i.e., the administrative structure of the country), and lack of cross-sectional coordination regarding “health-enhancing physical activity” were reported, highlighting scope for improvement when tackling PIA [28]. For men [6], Luxembourg showed reductions in the 2013–2017 period, but no changes in the period between 2002 and 2005. During those years, Luxembourg showed an improvement regarding local authority and local area perceptions offering opportunities to be physically active, pointing out a trend for reducing PIA prevalence [29]. Additionally, scientific efforts were made to understand the compliance of Luxembourgishs with physical activity recommendations and the potential demographic, socioeconomic, and perceptive factors influencing this behaviour at policy level, in order to inform decision-makers to target risk populations and develop preventive programs tacking physical inactivity [30].

Other changes were observed between periods when analyzing women and men separately in comparison with the study of Mayo et al. [11]: Firstly, Austria and Germany reduced the PIA prevalence in the period 2002–2005 but increased such prevalence in the period 2013–2017, that same pattern also occurring in Italian men. Secondly, Portugal’s PIA prevalence did not change between 2002 and 2005, yet increased between 2013 and 2017. This same pattern of change was also noted in Spanish men. Thirdly, France, Greece, Netherlands, and Sweden showed reductions in PIA prevalence for the period 2002–2005, yet no changes between 2013 and 2017 were observed. This same pattern was evident for Luxembourgs’ women and Belgium’s men. Lastly, Denmark, Finland, Ireland, and the United Kingdom did not report changes in PIA prevalence during either time frame, and this was also the pattern for women in Italy and Spain.

When comparing period changes in PIA prevalence between women and men, Belgium was the only country that showed gender differences in 2013 and then no gender differences in 2017. Some countries, such as Hungary, Latvia, Poland, and Slovenia eliminate gender differences between years without changes in the prevalence of women and men between years (i.e., no statistical differences were observed for those prevalence changes in women and men). On the other hand, countries such as Denmark, Estonia, Finland, Netherlands, and Sweden maintained no differences in gender PIA prevalence while reporting no changes between years.

Some countries showed an increase in prevalence in both sexes while maintaining no differences in the prevalence of women and men, such as Germany, Lithuania, Romania, or Slovakia. Bulgaria maintained no gender differences with increases men PIA prevalence. Other countries as Cyprus, France, Greece, Ireland, and the United Kingdom had gender differences at both time points with no increases in PIA prevalence between years. Malta and Spain maintained a gender differences with increases in women and men PIA prevalence, respectively. Lastly, Austria and Luxembourg changed from no gender differences of PIA prevalence in 2013 to reporting differences in 2017. In Austria, there was an increase in PIA for women, and in Luxembourg there was a decreasae in PIA prevalence for men. Croatia eliminated gender differences by increasing the PIA prevalence more in men than in women, while Czechia and Italy saw gender differences disappear through an increased PIA prevalence in women. Portugal maintained PIA prevalence gender differences while both genders reported PIA increases over time. These data show a limited gender-sensible approach while tackling PIA prevalence, particularly in the cases of Austria, Croatia, Czechia, Italy, Malta [6].

The previous study analyzing gender differences in PIA across 15 EU countries between 2002 and 2005 observed differences in both years with higher levels for women [11], which is consistent with our data. Nevertheless, we observed these gender differences after an increase of PIA, not a reduction. Interestingly, women in Belgium showed a decrease in PIA prevalence in both studies, which suggests a steady policy action for tackling PIA in women. This reduction occurred, nonetheless, despite not having suitable policy indicators of women physical activity participation in its national plan for the previous period [11, 31] or this particular one [26, 32].

Thus, this higher prevalence of PIA in women in comparison with men observed in our analysis is recurrent in the literature and consistent across countries [4–6] and timeline [4, 6], as data consistently show women participate in less leisure-time physical activity than men. To eliminate this systematic difference, more safe, accessible, and tailored activities are needed while changing cultural norms, traditional roles, and lack of social and community support. Only more rounded and sensible policies in which barriers are truly understood with structured policy delivery systems in place will help to eliminate or at least reduce the gender gap [4, 6].

The findings here should be considered in the light of some limitations. The differences in definitions, questionnaires, answers possibilities, methodological particularities, and means of analyzing data potentially limit reliable comparisons and the generalization of the findings [10]. In contrast, it is known that the IPAQ questionnaire tends to overestimate the physical activity reported [33]. That said, our data are broadly consistent with the literature and allow for tracking changes in PIA prevalence in any case. Future Eurobarometers should amend these differences in methodology, standardizing survey instruments to increase the comparability of the Eurobarometers, building up as a consequence better databases.

## Conclusions

The PIA prevalence increased in the overall EU sample between 2013 and 2017 and for both women and men separately. A higher prevalence of PIA was observed in women for both 2013 and 2017. Large differences were observed by country and year. Reductions in PIA prevalence were only for Belgium’s women and Luxembourg’s men. Increases in PIA prevalence were reported for women in Austria, Croatia, Germany, Lithuania, Malta, Portugal, Romania, and Slovakia, and for men in Bulgaria, Croatia, Czechia, Germany, Italy, Lithuania, Portugal, Romania, Slovakia, and Spain.

Between years, some countries eliminated gender differences without showing changes in PIA prevalence, such as Hungary, Latvia, Poland, and Slovenia. Other maintained similar gender rates of PIA prevalence while showing no changes over time, such as Denmark, Estonia, Finland, Netherlands, and Sweden. Overall, the changes noted in this study highlight limited success of gender-specific approaches to addressing PIA prevalence, while also suggesting no progress in reaching the 2025 target of a 10% reduction in PIA prevalence. The findings arising from this study should be used to help to strengthen the following policy action in the EU countries. Priorities in policy development should include defining the policy actions that are needed to progress in the accomplishment with the new PIA prevalence reduction goals for 2030 while, at the same time, reducing the gender disparities in PIA prevalence.

## Data Availability

The raw data is owned by the European Commission and available online (Special Eurobarometer 412, March 2014 [16]: https://dbk.gesis.org/dbksearch/sdesc2.asp?no=5877&search=Physical%20fitness%20and%20exercise&search2=&field=all&field2=&DB=e&tab=0&notabs=&nf=1&af=&ll=10. Special Eurobarometer 472, March 2018 [17]: https://dbk.gesis.org/dbksearch/sdesc2.asp?no=6939&search=Physical%20fitness%20and%20exercise&search2=&field=all&field2=&DB=e&tab=0&notabs=&nf=1&af=&ll=10).

## Abbreviations

CI: Confidence interval
EU: European Union
IPAQ: International Physical Activity Questionnaire
PA: Physical activity
PIA: Physical Inactivity
WHO: World Health Organization
